# Antioxidant and Antimicrobial Potencies of Chemically-Profiled Essential Oil from *Asteriscus graveolens* against Clinically-Important Pathogenic Microbial Strains

**DOI:** 10.3390/molecules27113539

**Published:** 2022-05-31

**Authors:** Mohammed M. Aljeldah

**Affiliations:** Department of Clinical Laboratory Sciences, College of Applied Medical Sciences, University of Hafr Al Batin, Hafar al-Batin 31991, Saudi Arabia; mmaljeldah@uhb.edu.sa

**Keywords:** medicinal plants, clinically important strains, pathogens, bioactive compounds, phytoconstituents

## Abstract

Recently, the antimicrobial potential of essential oils extracted from plants has gained extensive research interest, primarily for the development of novel antimicrobial treatments to combat emerging microbial resistance. The current study aims at investigating the antimicrobial activity and chemical composition of essential oil derived from gold coin daisy, which is known as *Asteriscus graveolens* (EOAG). In this context, a gas chromatography-tandem mass spectrometry (GC-MS) analysis of EOAG was conducted to identify its phytoconstituents. The in vitro antioxidant capacity of EOAG was determined by the use of three tests, namely: 1,1-diphenyl-2-picrylhydrzyl (DPPH), ferric reducing activity power (FRAP), and total antioxidant capacity (TAC). The antimicrobial activity of EOAG against clinically important bacterial (*Escherichia coli*, K12; *Staphylococcus aureus*, ATCC 6633; *Bacillus subtilis*, DSM 6333; and *Pseudomonas aeruginosa*, CIP A22) and fungal (*Candida albicans*, ATCC 10231; *Aspergillus niger*, MTCC 282; *Aspergillus flavus*, MTCC 9606; and *Fusarium oxysporum,* MTCC 9913) strains was assessed. Antimicrobial efficacy was determined on solid (inhibition diameter) and liquid media to calculate the minimum inhibitory concentration (MIC). GC/MS profiling of EOAG revealed that 18 compounds were identified, with a dominance of α-Thujone (17.92%) followed by carvacrol (14.14%), with a total identification of about 99. 92%. The antioxidant activity of EOAG was determined to have IC_50_ values of 34.81 ± 1.12 µg/mL (DPPH), 89.37 ± 5.02 µg/mL (FRAP), and 1048.38 ± 10.23 µg EAA/mg (TAC). The antibacterial activity in a solid medium revealed that the largest diameter was recorded in *P. aeruginosa* (28.47 ± 1.44 mm) followed by *S. aureus* (27.41 ± 1.54 mm), and the MIC in *S. aureus* was 12.18 ± 0.98 µg / mL. For the antifungal activity of EOAG, the largest inhibition diameter was found in *F. oxysporum* (33.62 ± 2.14 mm) followed by *C. albicans* (26.41 ± 1.90 mm), and the smallest MIC was found in *F. oxysporum* (18.29 ± 1.21 µg/mL) followed by *C. albicans* (19.39 ± 1.0 µg/mL). In conclusion, EOAG can be useful as a natural antimicrobial and antioxidant agent and an alternative to synthetic antibiotics. Hence, they might be utilized to treat a variety of infectious disorders caused by pathogenic microorganisms, particularly those that have gained resistance to standard antibiotics.

## 1. Introduction

Historically, medicinal plants have been extensively relied upon for their therapeutic potential, as they have been incorporated into ethnomedicinal practices dating back hundreds of years [1,2]. In addition to being intensively researched for their usefulness in medicine, plant derivatives have also received a lot of attention for their potential as growth and health promoters. Plant-derived substances have different uses; essential oils, in particular, have drawn special attention because of their widespread use as medicinal and food additives. Preventative and therapeutic medicines are increasingly using phytochemicals, especially those with anticarcinogenic and antibacterial properties [3].

Essential oils (EOs) are sustainably composed of volatile compounds, including terpenes, terpenoids, phenol-derived aromatic components, and aliphatic components with strong odors, and are generated as secondary metabolites by aromatic plants. EOs possess numerous biological properties, including antibacterial, analgesic, sedative, anti-inflammatory, spasmolytic, anesthetic, and antioxidant activities [4,5]. To date, these properties have not been altered much, except that more is now known about the modes of action, notably at the antimicrobial level [4].

Antioxidant agents serve a critical role in protecting the human body against several illnesses, including aging, cancer, neurological disease, and arteriosclerosis, as well as other pathological processes [6]. Increasing attention is being paid to plant-derived antioxidants, which might have a significant influence on health protection [7]. Users of food products tend to choose environmentally friendly antioxidants to prevent oxidative destruction from free radicals. Synthetic antioxidants, including tertbutyl hydroquinone, propyl gallate, and butylated hydroxyanisole, are no longer suggested because of their carcinogenic potential [8].

Antimicrobial resistance (AMR) is a phenomenon that happens when bacteria evolve techniques to resist antibiotics intended to kill them, resulting in infections that are difficult to cure and an increased risk of disease transmission [9]. Since it has emerged as one of the most significant challenges afflicting the healthcare system in recent decades, scientists have focused their attention more intensely on antibiotic resistance throughout the globe. AMR may emerge as a result of the overuse of medications in human medicine, animal husbandry, and hygiene [7,10,11]. AMR is a major cause for concern today, and if no new medications are produced to combat the underlying pathogens, the death toll might rise to 10 million by 2050, with substantial societal and economic ramifications [12]. 

Plant species have been extensively studied for their unique pharmacological properties [13], including the Asteraceae family, which is composed of approximately 25,000 species [14]. In this context, *Asteriscus graveolens* is one of the most popular species of this family that has been reported to possess a pharmacological potency. Hence, it has been widely employed in ethnomedicinal practices to treat various types of pathological conditions such as fever, digestive tract problems, and bronchitis [15]. EOs from *Asteriscus graveolens* possessed fungicidal properties towards *Alternari*a sp. and *Penicillium expansum* [16]. More is known about the EOs of *Asteriscus graveolens* growing under different climate and edaphic conditions, including anticancer activities, corrosion inhibition, antibacterial and antioxidant properties [17,18,19]. 

The chemical composition of EOs from the aerial parts of *Asteriscus graveolens* was previously profiled and was found to be rich in 6,7-dimethyl-l,5-hydroxy-3,5- octadiene, α-pinene, cedrenol, α-phellandrene, α-himachalene, 1,8-cineole, and T-cadinol [20,21]. 

The two-fold objectives of this study were: (*i*) to conduct a GC-MS chemical analysis to identify the phytoconstituents of EOAG and (*ii*) to investigate its antioxidant, antifungal and antibacterial properties by use of in vitro assays. 

## 2. Materials and Methods

### 2.1. Plant Material

Leaves of *Asteriscus graveolens* (Forssk.) Less. were collected in March 2021 from South El-Dahnaa desert, which is located along the road between Damam and Riyadh. The plant was identified by a botanist and was subsequently registered and deposited in the herbarium with the voucher number HR/AG-322. Prior to essential oil (EO) extraction by use of Clevenger apparatus, the leaves were air-dried in the laboratory under shaded conditions for 10 days and subsequently cut into a fine powder using an electric apparatus.

### 2.2. Extraction of EOAG

Around 200 g of the dry powder of *A. graveolens* was added to 750 mL of distilled water before being subjected to extraction by the use of a Clevenger-type apparatus at 100 °C for 3 h. The essential oils (EOs) were decanted, dried on Na_2_SO_4_, and saved far from light at 4 °C until further use.

### 2.3. Identification of Terpenic Compounds by GC/MS

#### 2.3.1. Gas Chromatography-Flame Ionization Detector (CG-FID)

The separated essential oils (EOAG) were diluted in hexane (10:100), and a sample of 1 µL was taken for gas chromatographic examination. The researchers employed a trace gas chromatograph (GC) (UTRA S/N 20062969, Thermo Fischer, Waltham, MA, USA) with an HP-5MS non-polar fused silica capillary column (60 m, 0.32 mm, film thickness 0.25 mL). Operating conditions: oven temperature program from 50 °C (2 min) to 280 °C at 5 C/min for 10 min; 2 “split mode” ratio 1:20; nitrogen (N2) carrier gas, flow rate 1 mL/min; injector and detector (flame ionization detector) temperatures were set to 250 °C and 280 °C, respectively.

#### 2.3.2. Gas Chromatography-Mass Spectrometry Analysis (GCMS)

The EOAG were analyzed using an HP-5MS non-polar fused silica capillary column on a Thermo Fischer capillary gas chromatograph immediately linked to a mass spectrometer system (model GC ULTRA S/N 20062969; Polaris QS/N 210729) (60 m, 0.32 mm, 0.25 mm film thickness). The following was the GC-MS oven temperature working condition: initial temperature 40 °C for 2 min, then 2 °C/min up to 260 °C using isotherm for 10 min; injector temperature 250 °C. The carrier gas was helium, which had a flow rate of 1 mL/min. The essential oils were diluted in hexane at a 10:100 ratio. The injection volume was 1 mL of diluted oil, split injection technique; ionization energy 70 eV, electronic ionization mode; ion source temperature 200 °C, scan mass range of *m*/*z* 40–650, and interface line temperature 300 °C. The retention indices (RI) of the components were calculated in comparison to those of a homologous series of n-alkanes (Fluka, Buchs/SG, Switzerland), and their recorded mass spectra were compared to those contained in the spectrometer database (NIST MS Library v. 2.0) and the literature [22].

### 2.4. Antioxidant Activity of EOAG

The in vitro antioxidant capacity of EOAG was assessed by three methods: DPPH (IC_50_), TAC, and FRAP (EC_50_). The antioxidant effectiveness of EOAG was determined by comparison with positive controls, BHT, and quercetin. 

#### 2.4.1. DPPH Test 

In the present work, 100 µL of EOAG at various concentrations (0.001 to 1 mg/mL) was mixed with 750 µL of DPPH previously prepared in methanol (0.004 %). After incubation at room temperature (RT) for 30 min, values of absorbance were recorded at 517 nm vs. a blank consisting of 750 µL of DPPH (at a final concentration of 0.004% in methnol) and 100 µL of methanol. The antioxidant effectiveness was evaluated by calculating the required concentration for the sample to scavenge 50% of the DPPH free radicals (IC_50_ in µg/mL). The percentage of inhibition (I%) was calculated based on the following equation:

$$I\%=\left[\frac{1-\mathrm{sample}}{\mathrm{control}}\right]\times 100\;$$

#### 2.4.2. FRAP Test 

The reducing power of EOAG was assessed by adding 1 mL of 0.2 M phosphate buffer (pH = 6.6) and 1 mL of 1% potassium ferricyanide to 0.2 mL of different concentrations of EOAG (0.001 to 1 mg/mL) in methanol (80%). Following incubation at 50 °C for 20 min, 1 mL of TCA (10%), 1 mL of dH_2_O, and 0.1% of 0.2 mL of FeCl_3_ were added. The absorbance of the reaction media was measured at 700 nm vs. a blank consisting of chemicals and methanol. The results are represented as the 50% effective concentration (EC_50_), which reflects the concentration of antioxidants needed to give an absorbance of 0.5 nm [23].

#### 2.4.3. TAC Test

Briefly, 2000 µL of H_2_SO_4_ solution (0.6 M), 28 mM sodium phosphate buffer, and 4 mM ammonium molybdate were mixed with 50 µL of EOAG (1 mg/mL). The reaction mixture and the blank were placed at 95 °C for 90 min in a water bath. Following cooling, the absorbance was recorded using a UV spectrometer at 695 nm [24,25]. The total antioxidant capacity (TAC) of EOAG was represented as micrograms of ascorbic acid equivalents per milligram of EOs (µg EAA/mg). 

### 2.5. Evaluation of the Antimicrobial Activity of EOAG

The antimicrobial activity of EOAG was assessed using pathogenic strains, including *Escherichia coli*, K12; *Staphylococcus aureus*, ATCC 6633; *Bacillus subtilis*, DSM 6333; and *Pseudomonas aeruginosa*, CIP A22, while fungal activity was tested vs. *Candida albicans*, ATCC 10231; *Aspergillus niger*, MTCC 282; *Aspergillus flavus*, MTCC 9606; and *Fusarium oxysporum*, MTCC 9913. Colonies of the microbial strains were suspended in sterile aqueous NaCl solution (0.9%), and the density of this microbial solution was adjusted to be almost 10^8^ CFU/mL [26]. The standard antibiotics kanamycin (0.1 mg/mL) and streptomycin (0.1 mg/mL) were utilized as references for comparison to EOAG.

#### 2.5.1. Disc Diffusion Method

The sensitivity of the microbial strains was evaluated using the disc diffusion technique, as described in previous work [27]. First, Petri plates (90 mm) containing Muller Hinton agar, yeast extract–peptone–glycerol, and potato dextrose agar media were inoculated with 1 mL of fresh microbial cultures before standing for 10 min. Following that, 6 mm sterile discs were impregnated with 10 µL of EOAG, and positive controls were placed on the Petri dishes. Finally, the plates were incubated at 37 °C for the bacteria strains, 30 °C for the yeast, and 27 °C for the molds for 24 h and 7 days. Following incubation, the zones around the wells were measured in millimeters.

#### 2.5.2. Determination of Minimum Inhibitory Concentration (MIC)

Briefly, various concentrations of EOAG were directly prepared in agar (0.2%). The dilutions of different microbial strains were performed as described in earlier work [27], by pouring 50 µL of inoculum into 96-well plates prior to incubation for 20 h at 37 °C. Next, the microbial growth was visualized by adding 10 µL of 2,3,5-triphenyltetrazolium chloride (TTC) (1%) to each well. Notably, the wells containing bacterial growth became pink due to the activity of the dehydrogenases, while the wells without bacterial growth remained colorless after 2 h of incubation. Therefore, the MIC was determined to be the lowest concentration showing no pink color [26]. 

### 2.6. Statistical Analysis

The findings presented in this research work are expressed as means with standard deviations of triplicate tests. The Shapiro–Wilks test was used to check normality, and Levene’s test was used to verify the homogeneity of variance. Tukey’s t test was employed as a post-hoc test for multiple comparisons. Statistical significance was considered at the cutoff of *p* ≤ 0.05. 

## 3. Results and Discussion 

### 3.1. GC-MS Profiling of EOAG

**Figure 1:**
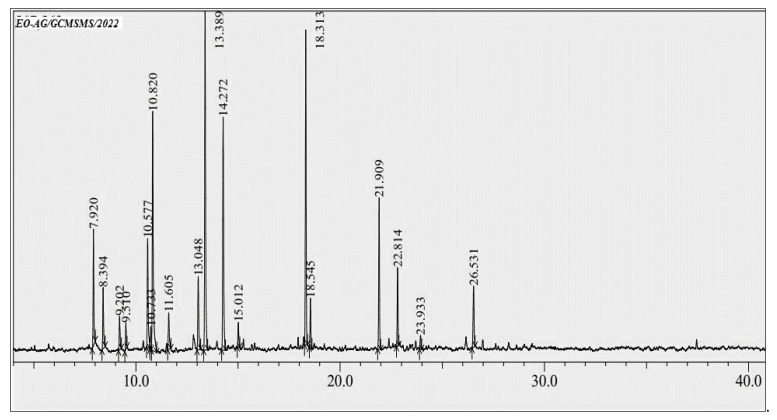
Chromatographic profile of EOAG by GC/MS.

**Figure 2:**
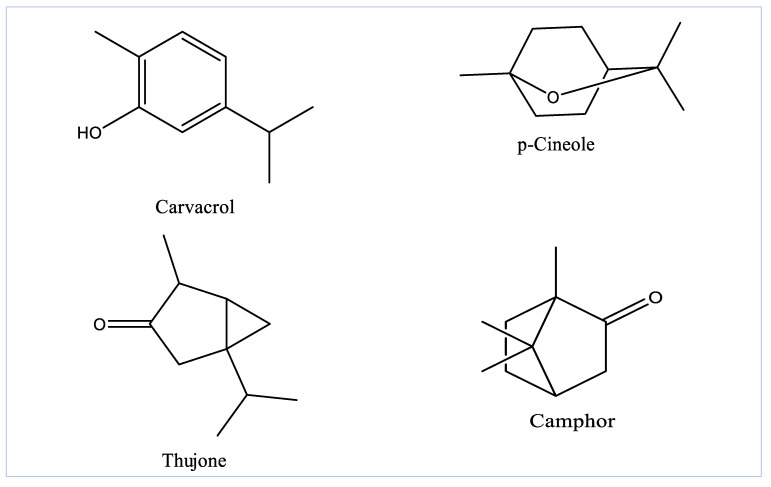
Molecular structures of the major compounds in EOAG.

The yield of EOAG obtained by the hydrodistillation of aerial parts of *A. graveolens* was 0.61%, which is higher than that of *A. graveolens* subsp. odorus growing elsewhere with a calculated value of 0.50% [25]. Moreover, the obtained EO yield is comparable to that reported by other EO-producing plants that have been widely exploited for EO production [26,27]. The analysis of EOAG by GC/MS revealed eighteen compounds constituting 99.92% of the total oil mass (Figure 1 and Table 1). EOAG is mainly composed of α-thujone (17.92%), carvacrol (14.14%), p-cineole (13.83%) and camphor (12.71 %) (Table 1 and Figure 2). Oxygenated monoterpenes (66.07%), monoterpene hydrocarbons (19.59%), and sesquiterpene hydrocarbons (10.22%) are predominant in EOAG (Table 1). These results are in accordance with the literature reporting that *A. graveolens* subsp. odorus is higher in oxygenated sesquiterpenes (56.05%) and oxygenated monoterpenes (53.9%) [25,28]. The results of EOAG chemical composition presented here agree with those reported by Cristofari and co-authors who showed that the aerial parts of *A. graveolens* were characterized by a high content of oxygenated sesquiterpenes with 6-oxo- and 6-hydroxycyclonerolidol as predominant components [29]. The difference in the chemical composition of EOs from *A. graveolens* growing in different geographical areas can be due to differences in the environmental and edaphic conditions predominant these areas, resulting in metabolic changes. In addition, the method employed in the extraction with solvents can also be responsible for differences [30,31].

### 3.2. In Vitro Antioxidant Activity of EOAG

**Figure 3:**
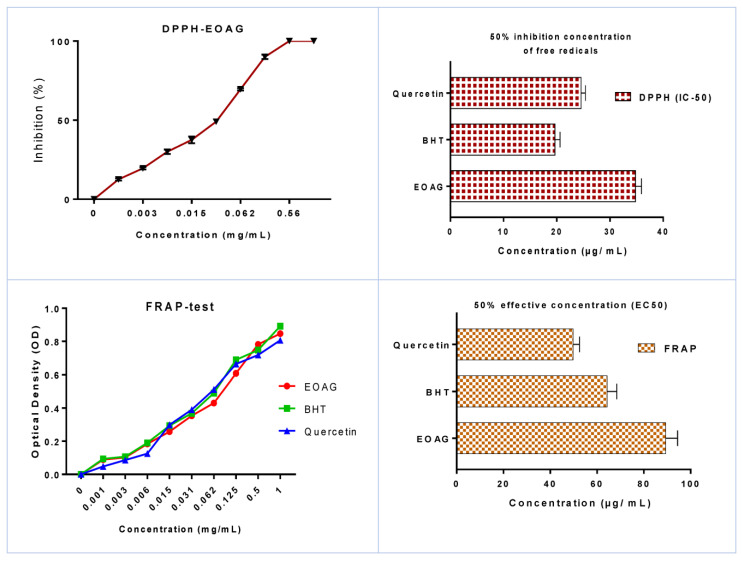
The antioxidant power of EOAG by the FRAP (EC_50_) and by DPPH (IC_50_) assays.

The antioxidant power, which was assessed by use of the DPPH method, revealed that EOAG exhibited potent antioxidant power in a dose–response association (Figure 3). Notably, inhibitions of free radicals on the order of 19.50 ± 0.71 %, 69.50 ± 0.70 %, and 89.17 ± 0.61 % were recorded for concentrations of 3 µg/mL, 60 µg/mL, and 100 µg/mL, respectively. The antioxidant efficiency of EOAG by DPPH is given in IC_50_, which was determined to be 34.81 ± 1.12 µg/mL. This value can be considered important when compared to those obtained with BHT and quercetin, which were used as positive controls and exhibited values of 19.68 ± 0.95 µg/mL and 24.57 ± 0.81 µg/mL, respectively (Figure 3). The antioxidant power of EOAG remains important when compared to that investigated by Alilou and co-authors [25], reporting an IC_50_ value of *A. graveolens* on the order of 249 µg/mL. The antioxidant potency of EOAG, as assessed by the DPPH assay, with an IC_50_ value of 34.81 ± 1.12 µg/mL is better than that found by Aouissi and co-authors who reported that the IC_50_ value of *A. graveolens* indigenous to Algeria was 420.16 mg/mL [18]. This difference can be due to differences in environmental and edaphic influences the plant growth, resulting in phytochemical composition changes, which in turn affect antioxidant power [31]. The results of the reducing power assay showed that EOAG recorded good antioxidant activity with an average EC_50_ value of EOAG of 89.37 ± 5.02 µg/mL, which is considered important when compared to standards BHT and quercetin, which registered EC_50_ values of 64.29 ± 4.15 µg/mL and 49.83 ± 2.69 µg/mL, respectively (Figure 3). 

**Figure 4:**
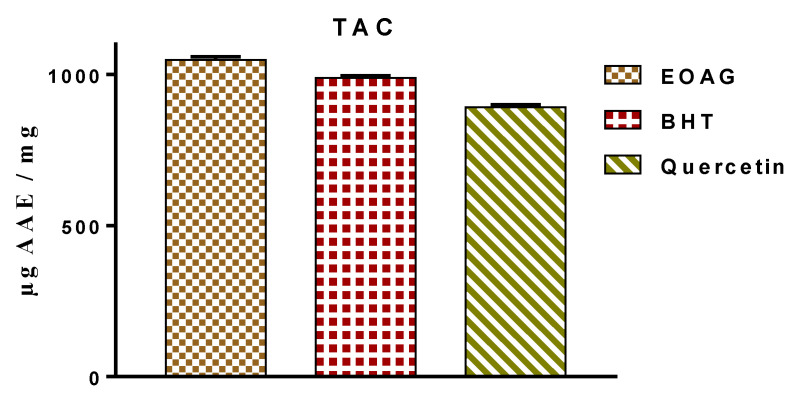
Total antioxidant capacity (TAC) determined by the ammonium phospho-molybdate assay.

The total antioxidant capacity (TAC) determined by ammonium molybdate method revealed that the antioxidant capacity of EOAG was on the order of 1048.38 ± 10.23 µg EAA/mg, while BHT and quercetin, which were used as chemical references recorded 987.46 ± 7.47 µg EAA/mg and 891.73 ± 8.22 µg/mg, respectively (Figure 4). These results are in accordance with those reported by Aouissi and co-authors, who determined the total antioxidant capacity of EOs of *A. graveolens* from Algeria was 0.28 AAEC/mg [18]. Previous literature reported that terpenic compounds, i.e., carvacrol and α-thujone, decreased the degree of peroxidation of the phospholipids present in liposomes in the presence of iron (III), which might explain the antioxidant power of EOAG in this study [32]. Scientific studies have shown that major compounds in EOs may be the potent agents responsible for the antioxidant power without excluding compounds detected in minute amounts, which may also react synergistically [33].

### 3.3. Evaluation of Antibacterial Activity of EOAG

**Figure 5:**
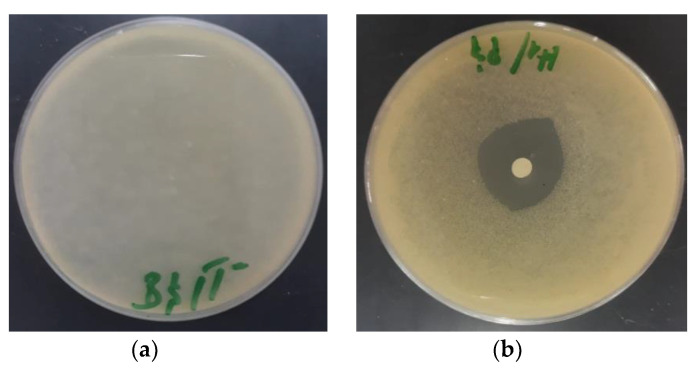

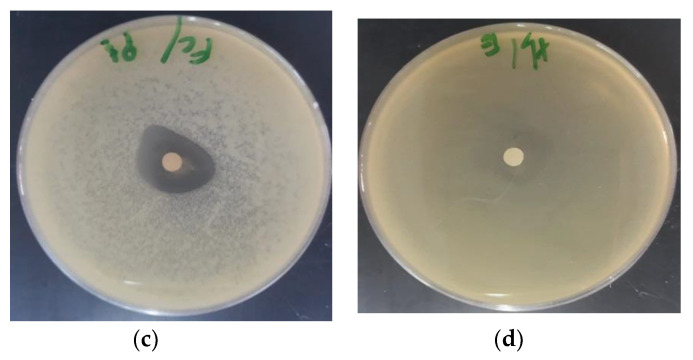
Photographs showing inhibition zones against bacteria. (**a**): negative control; (**b**): *B. subtilis*; (**c**): *E.coli*; (**d**): positive control.

The antibacterial potency of EOAG was determined by measuring the inhibition zone diameters and by determining the minimum inhibitory concentrations (MICs). The results showed that EOAG exhibited potent antibacterial effects vs. all tested bacteria (Table 2 and Figure 5). Notably, the largest inhibition diameter of EOAG was recorded for *P. aeruginosa*, which scored 28.47 ± 1.44 mm, followed by *S. aureus* with a calculated value of 27.41 ± 1.54 mm, whereas almost all bacteria showed resistance against kanamycin and streptomycin except for *S. aureus* and *E. coli* (Table 2 and Figure 5). Similarly, the MIC results showed that EOAG strongly inhibited the bacterial pathogenic strains used for testing, recording MICs values ranging from 12.18 ± 0.98 to 14.65 ± 1.28 µg/mL (Table 2).

Scientific studies have shown that the EOs with MICs ranging from 19 to 100 µg/mL are considered potent antibacterial agents [34]. It is thus fitting that our results are in agreement with this literature since the MIC values obtained with EOAG ranged from 12.18 ± 0.98 to 14.65 ± 1.28 µg/mL. The antibacterial results presented here agree with those found by Aouissi and co-authors who showed important antibacterial activity of Eos of *A. graveolens* from Algeria against *Escherichia coli, Klebsiella pneumonia, Salmonella typhi, Pseudomonas aeruginosa, Bacillus cereus,* and *Enterococcus faecalis* [18]. EOs are now well-recognized for their ability to combat hospital-acquired illnesses and pandemic multi-resistant bacteria [26,27,35]. The antibacterial capabilities and features of EOs can be explained by their lipophilic nature, which allows them to easily permeate the bacterial cell and eventually cause the bacterium’s death. Terpenes and hydrocarbons in EOs have been shown to preferentially react with the biological membranes of bacteria, resulting in membrane permeability disturbance, which ultimately leads to bacterial mortality [35,36]. More detailed scientific investigations on the mechanism of action of EOs containing hydrocarbon terpenes can help us better understand how they affect bacteria [37]. Our findings agreed with those reported in the literature linking the antibacterial potency of EOs to the relative proportions of camphor present [38], which proves that monoterpenes have antibacterial properties against a variety of microorganisms [39]. Terpene-rich EOs can easily cross bacterial cell walls and the cytoplasmic membrane, resulting in polysaccharide and phospholipid permeability disorders, which may lead to immediate bacterial death [40]. The molecular interaction of EO components with the bacterial membrane results in serious lesions, which might explain their antibacterial activity. It is also possible that compounds in EOs react in synergy or individually to have antibacterial effects [27]. 

### 3.4. Evaluation of Antifungal Activity of EOAG

The antifungal activity of EOAG, tested by use of the solid medium assay, showed that the studied oil exhibited a good antifungal effect vs. all fungi used in the experiment (Table 3). Remarkably, the largest inhibition zone diameters were recorded in *F. oxysporum,* with a calculated value of 33.62 ± 2.14 mm, followed by *C. albicans,* with a noticed value of 26.41 ± 1.90 mm. The results noted the antifungal resistance of *C. albicans* and *A. flavus* to fluconazole, which was used as a drug reference (Table 3). Similarly, the MIC results showed that EOAG strongly inhibited the fungal pathogenic strains used for testing, recording MICs values ranging from 18.29 ± 1.21 to 24.50 ± 1.30 µg/mL (Table 3).

Fungal infections are common in hospitalized patients around the world with several risk factors associated with a shortage of diagnoses [41]. Many epidemiological data on fungal infections reported that the fungal strains instigated in the present work are involved in a wide range of illnesses. Notably, Candida species have been shown to be responsible for nosocomial invasive fungal infections in hospitalized patients and are responsible for 8 to 10% of all nosocomial infections [35]. According to previous reports, invasive candidiasis is commonly associated with high fatality rates, and controlling these infections can be difficult because some antifungal treatments are no longer effective against resistant forms [27]. It is thus fitting that looking for an alternative treatment to control such infections is more warranted to combat AMR. Based on their results in combating pathogens, EOs can be investigated as an alternative treatment to control fungal infections [42]. In the present work, the major components in EOAG might act individually or synergistically in association with those present in minute quantities to inhibit the mycelial growth of fungal strains. The antifungal activity investigated in the present work can be attributed to α-thujone and carvacrol, which were revealed by GCMS in higher amounts in EOAG (Table 1) [31,35]. The antifungal results presented here are consistent with those found by Znini and co-authors who reported that EOs from *A. graveolens* possessed antifungal potency against postharvest phytopathogenic fungi in apples, namely, *Alternaria* sp. from the direct contact assay and *P. expansum* from the vapor assay tests [16]. Moreover, Aouissi and co-authors showed important antifungal activity of EOs of *A. graveolens* growing in Algeria against *Fusariumo.f. sp, Fusariumo.f. sp. Lycopercisi, Fusarium graminearum,* and *Fusarium culmorum* [18]. The toxicity of EOs against fungi might be attributed to EO terpenes and phenolic compounds, which are known to disrupt cell membranes, causing cellular material leakage and eventually causing microorganism death by inhibiting mitochondrial ATPase and the electron transport chain [35,43].

## 4. Conclusions

The present work investigates the chemical composition of EOs from the aerial parts of *Asteriscus graveolens* and their antioxidant, antibacterial, and antifungal properties. EOs were shown to have reasonable antioxidant and antimicrobial capabilities, which might be ascribed to the high concentrations of certain bioactive compounds such as α-thujone and carvacrol. The findings of the current research highlighted the advantages of EOAG as an effective eco-friendly agent with antioxidant and antimicrobial activities. Further research is thus required to assess the safety of these EOs as well as their non-target toxicity in both animals and humans.

## Figures and Tables

**Table 1:** Volatile compounds of EO identified by GC/MS.

	RT	Compound	Retention Index	Chemical Class	Area (%)
1	7.92	α-Pinene	938	MO.H	5.10
2	8.39	Camphene	949	MO.H	2.91
3	9.20	β-Pinene	974	MO.H	1.58
4	9.51	Myrcene	988	MO.H	1.16
5	10.57	o-Cymene	1022	MO.H	5.70
6	10.73	Limonene	1028	MO.H	1.16
7	10.82	p-Cineole	1039	MO.O	13.83
8	11.60	γ-Terpinene	1058	MO.H	1.98
9	13.04	Isothujone	1002	MO.O	3.98
10	13.38	α-Thujone	1102	MO.O	17.92
11	14.27	Camphor	1141	MO.O	12.71
12	15.01	Borneol	1168	MO.O	1.23
13	18.31	Carvacrol	1297	MO.O	14.14
14	18.54	Thymol acetate	1357	MO.O	2.26
15	21.90	Caryophyllene	1404	ST.H	6.71
16	22.81	α-Humulene	1459	ST.H	3.51
17	23.93	Eugenol acetate	1525	O	0.61
18	26.53	Pogostol	1651	ST.O	3.43
		**Chemical Class**			
		Monterpene oxygenated (MO.O)			66.07
		Monoterpene hydrocarbons (MO.H)			19.59
		Other (O)			0.61
		Sesquiterpene oxygenated (ST.O)			3.43
		Sesquiterpene hydrocarbons (ST.H)			10.22
		Total			99.92%

RT; Retention time; MO.O: Monoterpene oxygenated; MO.H: Monoterpene hydrocarbons; O: Other; ST.O: Sesquiterpene oxygenated; ST.H: Sesquiterpene hydrocarbons.

**Table 2:** Antibacterial power of EOAG as assessed by use of inhibition zone diameters and MIC assays.

		*S. aureus*	*E. coli*	*B. subtilis*	*P. aeruginosa*
EOAG	Inhibition diameter (mm)	27.41 ± 1.54 ^a^	19.68 ± 1.25 ^b^	17.48 ± 1.75 ^b^	28.47 ± 1.44 ^a^
	MIC (µg/mL)	12.18 ± 0.98 ^b^	14.57 ± 0.87 ^b^	22.48 ± 0.69 ^a^	14.65 ± 1.28 ^a^
Stp	Inhibition diameter (mm)	10.73± 0.45 ^a^	0 ^b^	0 ^b^	0 ^b^
	MIC (µg/mL)	15.83 ± 0.20 ^a^	0 ^b^	0 ^b^	0
Kan	Inhibition diameter (mm)	0 ^b^	17.24 ± 1.34 ^a^	0 ^b^	0 ^b^
	MIC (µg/mL)	0 ^b^	13.47 ± 0.92 ^a^	0 ^b^	0 ^b^

Row values with different letters differed significantly (one-way ANOVA; Tukey test, *p* < 0.05). MIC: minimum inhibitory concentration; *Staphylococcus aureus* ATCC 6633; *Escherichia coli* K12; *Bacillus subtilis* DSM 6333; *Pseudomonas aeruginosa* CIP A22; Stp: Streptomycin; Kan: Kanamycin.

**Table 3:** Antifungal power of EOAG as assessed by use of inhibition zone diameters and MIC assays.

		*C. albicans*	*A. niger*	*A. flavus*	*F. oxysporum*
EOAG	Inhibition diameter (mm)	26.41 ± 1.90 ^a^	17.01 ± 1.08 ^b^	16.76 ± 1.02 ^b^	33.62 ± 2.14 ^c^
	MIC (µg/mL)	19.39 ± 1.0 ^a^	24.50 ± 1.30 ^b^	23.74 ± 1.81 ^b^	18.29 ± 1.21 ^a^
Flu	Inhibition diameter	0 ^a^	11.41 ± 1.31 ^b^	0 ^a^	16.18 ± 2.43 ^c^
	MIC (µg/mL)	0 ^a^	10.27 ± 0.84 ^b^	0 ^a^	33.12 ± 1.38 ^c^

Row values with different letters differed significantly (one-way ANOVA; Tukey test, *p* < 0.05). MIC: minimum inhibitory concentration; Staphylococcus aureus ATCC 6633; Escherichia coli K12; Bacillus subtilis DSM 6333; Pseudo-monas aeruginosa CIP A22; Stp: Streptomycin; Kan: Kanamycin; R: Resistant; C.A: *Candida albicans ATCC 10231; Aspergillus niger MTCC 282; Aspergillus flavus MTCC 9606; Fusarium oxysporum MTCC 9913; Flu: Fluconazole.*

## Data Availability

All data reported here are available from the authors upon request.

## Sample Availability

Samples of EOAG are available upon request from the author.
